# Prognostic factors and outcomes of COVID-19 cases in Ethiopia: multi-center cohort study protocol

**DOI:** 10.1186/s12879-021-06652-0

**Published:** 2021-09-16

**Authors:** Saro Abdella, Masresha Tessema, Geremew Tasew, Atkure Defar, Asefa Deressa, Feyisa Regasa, Frehiwot Teka, Eyasu Tigabu, Dereje Nigussie, Tefera Belachew, Million Molla, Amare Deribew, Workeabeba Abebe, Tegbar Yigzaw, Tsinuel Nigatu, Getnet Mitike, Tewodros Haile, Haftom Taame, Muhammed Ahmed, Frehiwot Nigatu, Tola Tolesa, Eskinder Wolka, Wondwossen Amogne, Arnaud Laillou, Misker Amare, Yaregal Fufa, Alemayehu Argaw, Woldesenbet Waganew, Akilili Azazh, Aschalew Worku, Berhane Redae, Menbeu Sultan, Miraf Walelegn, Muluwork Tefera, Sisay Yifru, Rahel Argaw, Natinael Brehau, Sisay Teklu, Getachew Demoz, Yakob Seman, Mihretab Salasibew, Eshetu Ejeta, Susan J. Whiting, Dawit Wolday, Getachew Tollera, Ebba Abate, Dereje Duguma

**Affiliations:** 1 Ethiopian Public Health Institute, Addis Ababa, Ethiopia; 2 COVID-19 Isolation and Treatment Center, Eka Kotebe General Hospital, Addis Ababa, Ethiopia; 3 Saint Paul’s Hospital, Addis Ababa, Ethiopia; 4 International Institute for Primary Health Care, Addis Ababa, Ethiopia; 5 Mekelle University, Mek’ele, Ethiopia; 6 Jimma University, Jimma, Ethiopia; 7 Saint Peter Hospital, Addis Ababa, Ethiopia; 8 Nutrition International, Addis Ababa, Ethiopia; 9 Addis Ababa University, Addis Ababa, Ethiopia; 10 Jhpiego-Innovating, Addis Ababa, Ethiopia; 11 Harvard T.H. Chan School of Public Health, Boston, MA USA; 12 Africa CDC, Addis Ababa, Ethiopia; 13 UNICEF, Addis Ababa, Ethiopia; 14 Children’s Investment Fund Foundation, London, UK; 15 College of Pharmacy and Nutrition, University of Saskatchewan, Saskatoon, SK Canada; 16 Ethiopian Ministry of Health, Addis Ababa, Ethiopia

**Keywords:** COVID-19, Prognostic Factors, Outcomes of COVID, Cohort, Ethiopia

## Abstract

**Background:**

The coronavirus disease 2019 (COVID-19) is caused by severe acute respiratory syndrome coronavirus (SARS-CoV-2) and became pandemic after emerging in Wuhan, China, in December 2019. Several studies have been conducted to understand the key features of COVID-19 and its public health impact. However, the prognostic factors of COVID-19 are not well studied in the African setting. In this study, we aim to determine the epidemiological and clinical features of COVID-19 cases, immunological and virological courses, interaction with nutritional status, and response to treatment for COVID-19 patients in Ethiopia.

**Methods:**

A multi-center cohort study design will be performed. Patients with confirmed COVID-19 infection admitted to selected treatment centers will be enrolled irrespective of their symptoms and followed-up for 12 months. Baseline epidemiological, clinical, laboratory and imaging data will be collected from treatment records, interviews, physical measurements, and biological samples. Follow-up data collection involves treatment and prognostic outcomes to be measured using different biomarkers and clinical parameters. Data collection will be done electronically using the Open Data Kit (ODK) software package and then exported to STATA/SPSS for analysis. Both descriptive and multivariable analyses will be performed to assess the independent determinants of the treatment outcome and prognosis to generate relevant information for informed prevention and case management. The primary outcomes of this study are death/survival and viral shedding. Secondary outcomes include epidemiological characteristics, clinical features, genetic frequency shifts (genotypic variations), and nutritional status.

**Discussion:**

This is the first large prospective cohort study of patients in hospitals with COVID-19 in Ethiopia. The results will enable us to better understand the epidemiology of SARS-CoV-2 in Africa. This study will also provide useful information for effective public health measures and future pandemic preparedness and in response to outbreaks. It will also support policymakers in managing the epidemic based on scientific evidence.

*Trial Registration:* The Protocol prospectively registered in ClinicalTrials.gov (NCT04584424) on 30 October, 2020.

## Background

On March 11, 2020, the World Health Organization (WHO) declared Corona Virus Disease (COVID-19) to be a pandemic [1]. The current new emerging virus is a different strain of virus from SARS and MERS CORONA viruses. The difference is not only limited to genetic makeup but also in the clinical presentations, case fatality, and the rate of spread across the globe (surveillances). The swift spread of the virus is largely attributed to its stealth transmission, for which infected patients may be asymptomatic or exhibit only flu-like symptoms in the early stage. These undetected transmissions present a remarkable challenge for the containment of the virus and pose an appalling threat to public health [2]. COVID-19 has become one of the leading causes of mortality around the world.

As of August 03, 2021, there have been 200,508,148 confirmed cases and 4.26 million deaths reported worldwide and nationally 281,300 confirmed cases and 4,395 deaths reported [3]. To this end, the COVID-19 Global Research and Innovation Forum recommended each country and region to conduct research and generate evidence in their respective local context to strike the right balance between stopping transmission now and prepare for the future [4].

COVID-19 has also sparked fears of an impending economic crisis and recession [5]. Social distancing, self-isolation and quarantine, travel restrictions and border shut downs have led to a reduced workforce across all economic sectors and caused many jobs to be lost. Schools have been closed down, and the need for commodities and manufactured products has decreased [6]. Along with its high infectivity and fatality rates, there has been mass fear of COVID-19, termed as “coronaphobia”, that has generated a plethora of psychiatric manifestations across the different strata of the society [7]. The COVID-19 itself, multiplied by forced quarantine to combat the disease applied by nationwide lockdowns can produce acute panic, anxiety, obsessive behaviors, hoarding, paranoia, depression, and post-traumatic stress disorder (PTSD) [7, 8].

Several socio-demographic, clinical, immunological and nutritional factors are associated with COVID-19 disease progression. Evidence shows that COVID-19 disease progression is worse in people with suppressed immunity due to old age, pre-existing health conditions [9], and genetic conditions [10]. However, a comprehensive understanding of the underlying immunological process, particularly within cellular immunity, is still not well defined. The clinical disease spectrum of the SARS COV-2 ranges from subclinical or manifesting as unremarkable respiratory symptoms to severe respiratory complications such as pneumonia and respiratory distress.

There is growing literature highlighting obesity as a significant risk factor for the development of severe COVID-19. Body mass index (BMI) is an anthropometric measure used to assess obesity risk and has been observed as a risk factor in MERS-CoV [11] and influenza [12]. A recent systematic review showed that high BMI plays a significant role in COVID-19 severity in all ages, especially in the elderly population [13]. Obesity is associated with increased severity and mortality in pandemic H1N1 influenza and other respiratory viruses [14].

With the currently available evidence so far, children contribute 1–5% of total COVID-19 cases worldwide [15]. Asymptomatic disease in SARS-CoV-2 positive children ranged from 13% in China to 21% in Italy. Clinical features in symptomatic children are generally milder than adults; a very small proportion of those aged under 19 years have developed severe (2.5%) or critical disease (0.2%) [16].The USA and Chinese data showed bimodal age distribution among critically ill children, infants and adolescents > 15 years were more likely to be hospitalized due to COVID-19, and death was extremely rare with only a few numbers of reported cases [17].

Evidence on the effect of COVID-19 on pregnancy and its outcomes seems to be controversial. In some studies, pregnant mothers with confirmed COVID-19 infection were discharged without any major complications after giving birth. However, severe maternal morbidity resulting from COVID-19 and perinatal deaths has been documented in other studies [18]. There is also limited evidence about in utero infection and early positive neonatal testing. For example, a prospective cohort study conducted among pregnant women admitted to the hospital with confirmed SARS-CoV-2 infection in the UK revealed that 12 (5%) of 265 infants tested positive for SARS-CoV-2 RNA, six of them within the first 12 h after birth [19]. Contrary to this, infection has not been found in neonates delivered from pregnant women with COVID-19 based on a report from Hubei Province, China [20]. Further research, therefore, is required to determine the effect of COVID-19 on pregnancy and birth outcomes in our situation.

Nutrition is a key determinant of health [21]. More importantly, it is part of the treatment regimen for acute and chronic diseases and applies particularly to ailments for which an etiologic treatment has not yet been discovered and validated. An adequate diet is necessary to provide energy for the body’s functions and nutrients to build and repair tissues, prevent sickness, and help the body heal from illness [22]. A balanced diet also has a vital role in bolstering the immune response of an infected person against RNA viral infections [23]. There is sufficient evidence to demonstrate that the immune response can be weakened by inadequate nutrition [22, 23, 25]. The role of some nutrients in immune function and infectious diseases is well established, such as vitamin D [25].

In recent studies, COVID-19 is shown to be commonly complicated with coagulopathy, and the management of thromboembolism has significant importance in reducing mortality and morbidity [26, 27]. This indicates that a severe cytokine storm induces SARS-Cov-2 to lead to the coagulation cascade, causing thromboembolism, which is linked to abnormal parameters such as increases in fibrin, fibrin degradation products, fibrinogen, and D-dimer. Furthermore, immobility, systemic inflammation, platelet activation, endothelial dysfunction, and stasis of blood flow have been reported as the predisposing factors of thromboembolism [27]. The WHO recommended the use of low molecular weight heparin (e.g., enoxaparin), according to local and international standards, to prevent venous thromboembolism, when not contraindicated in COVID-19 patients [28]. However, the incidence of venous thromboembolism in COVID-19 patients hospitalized and under thromboprophylaxis is unclear.

To understand the negative impacts of COVID-19 on public health and key features pertinent to the disease, various studies are under investigation at the global level and they are contributing to delineating the characteristics of the disease and its lethality. Currently, it is recognized that a ‘one size fits all’ approach towards the design and implementation of interventions may not be appropriate. Therefore, global priorities, protocols, and intervention assessments have to be contextualized and adjusted to local needs and realities, including the translation of results. Therefore, this study aimed to determine the natural history of the disease; clinical features and management, epidemiological characteristics, immunological, virological courses and treatment response of the disease; effects of diets on the nutritional status on disease progression; effect of the disease on pregnancy and birth outcomes; and, the effect of traditional/modern treatment on COVID-19 outcome among patients admitted to treatment centers in Ethiopia.

## Methods/design

### Aim and study setting

In this multi-site cohort study, we aimed to determine the natural history of the disease; clinical features and management, epidemiological characteristics, immunological, virological courses and treatment response of the disease, effects of diets on the nutritional status on the disease progression, effect of the disease on pregnancy and birth outcomes, and the effect of traditional/modern treatment on COVID-19 outcomes among patients admitted to treatment centers in Ethiopia. Therefore, the study findings will generate scientific data for a systematic understanding of natural history, epidemiological characteristics, clinical features, and management of COVID-19 which will, in turn, enable the country’s health sector to develop strategies to prevent and control the pandemic before it poses further health and socioeconomic crises. The research question of this study is “Do different individual host factors and environmental situations influence clinical, epidemiological, and viral outcomes of COVID-19 infection?”.

The study will be conducted in all Federal treatment centers, including Ekakotebe, Yekatit-12, St. Peter Specialized hospitals, SPHMMC, Millennium hall center in Addis Ababa, and Regional Hospitals including Mekelle, Semera, Bahir Dar, Adama/Mojo, Hawassa, Jijiga, Harar, DireDawa, Metekel or Benishangul Gumuz, and Gambela dedicated to COVID 19 treatment.

### Study design and period

A multi-center prospective open cohort study design on COVID-19 confirmed cases in Ethiopia conducted from December 01, 2020 to December 2021. Like many other disease-specific general open cohorts, such as in Framingham Heart Study and the Ethiopia Netherlands AIDS Research Project (ENARP) studies, the study intends primarily to measure the incidence of several epidemiological, clinical, virological, and immunological outcomes of COVID-19 cases [29, 30]. The cohort will be an unbiased extensive routine collection of clinical, radiographic, laboratory, and clinical management, virological, immunological and nutritional data, which helps to detect and address emerging research priorities without relying on a priori hypothesis.

### Study population

This study will enroll individuals with confirmed infection with COVID-19 at selected Federal and Regional Hospitals irrespective of their differences (age, sex, symptoms, severity, and any other conditions). The patients' follow-up will be done strictly within the management adapted to their infection.

Individuals/patients in the study hospital or area will be eligible for inclusion if they meet the following criteria: (1) patient is admitted to selected treatment centers (Federal and Regional’s Hospitals) with COVID-19 confirmed by RT-PCR; and (2) consents to be enrolled in the follow-up study and provide all necessary information/data, blood sample, and nasopharyngeal swab for testing. Patients will be excluded if: (1) a subject deprived of freedom, subject under a legal protective measure; (2) refusal by a participant, parent or appropriate guardian or representative; (3) not willing to stay 12 months in the cohort in Ethiopia; (4) is already involved in the COVID-19 clinical trial or other interventional studies; (5) not capable of understanding or complying with the study protocol or provide consent; (6) anticipated transfer to another hospital that is not a study site within 72 h.

### Sample size and sampling procedures

The estimated sample size for this study is 6,390, based on the assumptions of a 28 percent death rate from a retrospective study, a design effect of 2.5, and a 20 percent loss to follow-up. A baseline evaluation or assessment will be performed immediately after screening. After baseline assessment, all patients will be followed-up daily until discharge according to WHO and National discharging guideline and followed as per schedule after discharged. Study subjects enrolled will be followed up for 12 months after enrollment for the specific objective related to virology and immunology, clinical course of the disease, response to treatment, effect of COVID-19 in pregnant mothers and birth-related effects of COVID-19. Enrolled cases will be followed up at 2 weeks, 3 months, 6 months, 9 months, and 12 months post-discharge. A visit window of ± 7 days may be applied to these visits. Patients discharged after recovery will be followed up according to the follow-up schedule.

### Primary outcome and secondary outcomes

The primary outcome variables are treatment outcome (active cases, recovered, death or transferred for further treatment), recovery time, and duration of viral shedding (the time from the first positive RT-PCR results to the occurrence of the last positive RT-PCR results or negative RT-PCR results). The secondary outcomes include clinical symptoms and signs (major); co-morbidities; status at last follow-up (survival, severity, virus detection); laboratory biomarkers; duration of symptomatic phase, inpatient stay, ICU stay; viral loads; anti-SARS-CoV-2 antibody titer; imaging with results; therapeutic measures; plasma 25-hydroxyvitamin D concentrations; dietary history; micronutrient status (zinc and vitamin A); fasting blood sugar; weight, height, BMI; total cholesterol (TC), triglycerides (TG), HDL-C, LDL-C and body composition(waist circumference); genetic frequency shifts (genotypic variations); obstetric and gynecologic history; pregnancy status/test; clinical status; supplemental oxygen; non-invasive ventilation or oxygen delivery devices/ respiratory support. We will also collect confounding variables include socio-demographic variables (age, sex, education status); status cases at the time of enrollment; and months after the first positive test.

### Data collection

Data collection will be done electronically using the REDCap software package and data documentation will be performed from a retrospective history of the patient and prospectively after treatment is finalized. Contact information such as home address and telephone number will be collected to facilitate follow up and for tracking defaulters of follow-up. For asymptomatic cases whose follow-up is undertaken at home, their home address, GPS coordinates and geospatial data for all participants will also be registered by trained data collectors (study nurse or doctor). Data will be gathered from patients and follow up cases through interviews by physicians/nurses at each treatment center using a questionnaire and standardized Case report form (CRF). Biological samples will also be collected at baseline and follow up when samples are taken in the context of care to meet the research objectives. Five-milliliter venous blood will be collected using a serum separator tube and 4 mL venous blood will be collected using a test tube containing anticoagulant. Viral load measures in a body fluid using Abbot and Rosh reagents and platforms will be measured. Phylogenetic analysis of SARS-CoV-2: genotyping of different types of SARS-CoV-2 from Ethiopian isolates under different settings will be done using a next-generation sequencer at Ethiopian Public Health Institute (EPHI). Imaging: chest x-ray, ultrasound scan, chest computed tomographic (CT) scans data will be retrieved if available. Laboratory tests for D-dimers, platelet count, PT, PTT, INR, D- Dimer, and serum ferritin will be measured.

The screening will be performed within the period between one to two days of admission to the hospital to review study inclusion/exclusion criteria; obtain prior medical and concomitant medication histories; perform a complete physical examination, including height, body weight, and vital signs (blood pressure, BP, heart rate, HR), Oxygen saturation (SpO2) with pulse oximetry; and collect a blood sample for laboratory testing on screening day.

### Baseline assessment

The baseline assessment (day 1) will be scheduled immediately after screening. At baseline, the following tasks will be performed:

1. Review and record all prior workup or test results.
2. Record detailed history of prior medication use in the subject.
3. The query for signs and symptoms and updated medications used since the screening visit. Any signs or symptoms reported after study drug administration should be recorded as adverse events. Any signs or symptoms reported before the study should be recorded as a pre-existing condition but should be collected and recorded in detail.
4. Perform a complete physical examination.
5. Obtain vital signs (HR, BP, RR, Oxygen saturation, capillary refill), Oxygen saturation (SpO2) with pulse oximetry.
6. Obtain body height, weight, and waist circumference.
7. Obtain the dietary history of the patients.
8. Collect urine for urinalysis, with the microscopic exam and for a pregnancy test (in women).
9. Collect blood for CBC and for serum chemistries including creatinine, total bilirubin, albumin, ALT, AST, total protein, CK, Creatinine Clearance (CrCl), and Ab assays, fasting blood sugar, lipid profiles (TC, LDL, HDL and triglycerides), plasm 25-hydroxy vitamin D concentration, laboratory test for D-dimers, platelet count, PT PPT, INR, and serum ferritin.
10. Collect serum (recommend 5 mL serum storage at the investigational site as a back-up specimen).

### Follow-up visits

Data collection at follow-up at 2 weeks, 3 months, 6 months, 9 months and 12 months was the same as at baseline and used to ascertain primary and secondary outcomes. The following tasks will be performed at these visits:

- Perform symptom-Directed physical exam,
- Obtain body weight and vital signs including pulse, respiratory rate and blood pleasure, oxygen saturation,
- Perform pulmonary function test (PFT),
- Record any symptoms,
- Spirometry to determine pulmonary function tests such as forced expiratory volume in the first second (FEV1), forced vital capacity (FVC) and FEV1/FVC ratio,
- Collect blood for serum chemistries including creatinine, electrolytes, total bilirubin, albumin, ALT, AST, total protein, CK, calculate CrCl, Ab tests,
- Collect mouth/nasal swabs for PCR tests,
- Collect urine for analysis, including pregnancy test (in women),
- Collect serum on-site as the back-up storage (3 mL serum) and
- Collect dietary history.

### Data processing and statistical analysis

Data collection will be done electronically using the ODK software package and then exported to STATA/SPSS for data cleaning, management, and statistical analysis. The data will be cleaned and checked for outliers, all assumptions, and analyzed using appropriate statistical software (STATA, Python, and/or R statistic software).The analysis will be performed by trained personnel (epidemiologists, statisticians, health economists) using the appropriate statistical methods. Statistical analyses strategy will be based on specific study objectives and the nature of the outcome variables, the various assumptions to be assessed and associated scenarios to be considered, and the criteria to be used for decision-making at each stage including management of incomplete data, model building procedures, and evaluating the validity of models and the robustness of results. Data will be assessed for consistency, missing values, presence of outliers and implausible values, and different statistical assumptions including univariate and multivariate normality of distributions, the linearity of hypothesized relationships, the proportionality of hazard, and the presence of rare events among others.

A descriptive summary of the continuous variables will be reported as mean (± SD) and median (IQR), and categorical variables will be summarized as counts and percentages. A correlation or chi-square test will be performed for appropriate variables.

Predictors that have been identified as risk factors in the bivariate analysis will be entered into multivariable analysis to isolate independent determinants of the outcome. Multiple linear and generalized linear regression models will be fitted depending on the type of outcome variable. Cox-proportional hazard ratio (HR) will be used for time-to-event variables including the time to recovery, and other time to event variables. Time to event analysis, semi and parametric survival analysis will be conducted as appropriate.

To take into account the multi-center cohort design, i.e., clustering of data by treatment centers and repeated measurements per subject over time, a multilevel modeling approach will be employed using mixed-effects modeling or generalized estimating equation. Incomplete data (missing values and lost-to-follow-up cases) will be handled using model-based approaches, such as full information maximum likelihood estimation and multiple imputation modeling after the missing data mechanisms and assumptions are evaluated. Regression models will be evaluated for potential problems including collinearity among predictors, omitted variables bias, and model over-adjustment (endogeneity). The final fitted models will be assessed for validity and robustness of results on a subsample of data not used in the model-building procedure. For this purpose, a random subsample from the original data will be kept before starting the analysis. Statistical significance will be considered at α < 0.05. However, to control for the inflated probability of Type-I error due to multiple hypotheses testing, P-values will be adjusted for false discovery rate using the Benjamini–Hochberg method. The results will be described as percentages, tables, figures, and association significance.

## Discussion

SARS-COV-19 pandemic has caused huge detrimental impact in social interaction and economic aspects globally to an extent unseen before. Moreover, the healthcare systems have become overloaded even in developed countries. This is the first large prospective cohort study of patients in hospitals with COVID-19 in Ethiopia. The results will enable us to better understand the epidemiology of SARS-CoV-2 in an African setting. This study will also provide useful information for effective public health measures and future pandemic preparedness and in response to outbreaks. It will also support policymakers in managing the epidemic based on scientific evidence.

## Data Availability

As this study is not started, data are not available at this time. De-identified data will be available after publication of primary analyses, upon communication with the corresponding authors and according to the requirements of applicable IRBs and institutional policies.

## Abbreviations

COVID-19: Coronavirus Disease 2019
SARS-CoV: SARS Coronavirus
MERS-CoV: Middle Eastern Respiratory Syndrome Coronavirus
H1N1: Influenza A Virus Subtype H1N1
RNA: Ribonucleic Acid
SPHMMC: Paul's Hospital Millennium Medical College
RT-PCR: Reverse Transcription Polymerase Chain Reaction
ICU: Intensive Care Unit
GPS: Global Positioning System
CT: Chest Computed Tomographic
PT: Prothrombin Time
PTT: Thromboplastin Time
INR: International Normalized Ratio
HR: Heart Rate
BP: Blood Pressure
SpO2: Oxygen Saturation
RR: Respiratory Rate
CBC: Complete Blood Count
ALT: Alanine Aminotransferase
CK: Creatine Kinase
AST: Aspartate Aminotransferase
CrCl: Creatinine Clearance
FEV1: First Second of the Forceful Exhalation
FVC: Forced Vital Capacity
IQR: Inter Quartile Range
COVID: Corona Virus Disease
BMI: Body Mass Index
EPHI: Ethiopian Public Health Institute
rRT-PCRS: Reverse Transcription Real Time Polymerase Chain Reaction
FVC: Forced Vital Capacity
PFT: Perform Pulmonary Function Test
CRF: Standardized Case Report Form
TC: Total Cholesterol
LDL: Low-density Lipoprotein
PTSD: Post-traumatic Stress Disorder
ENARP: Ethiopia Netherlands AIDS Research Project
SARS-CoV-2: Severe Acute Respiratory Syndrome Coronavirus
ODK: Open Data Kit
TG: Triglycerides
HDL-C: High-density Lipoprotein Cholesterol
LDL-C: Low-density Lipoprotein Cholesterol
